# Dexmedetomidine or Butorphanol for Co-Induction of General Anaesthesia with Propofol in Unpremedicated Healthy Dogs: Clinical and Echocardiographic Assessment

**DOI:** 10.3390/vetsci12090885

**Published:** 2025-09-13

**Authors:** Giuliano Ravasio, Martina Amari, Chiara Locatelli, Francesco Ferrari, Andrea Jacchetti, Valerio Bronzo, Federica Alessandra Brioschi

**Affiliations:** 1 Department of Veterinary Medicine and Animal Sciences (DIVAS), Università degli Studi di Milano, Via dell’Università 6, 26900 Lodi, Italy; giuliano.ravasio@unimi.it (G.R.); francesco.ferrari@unimi.it (F.F.); valerio.bronzo@unimi.it (V.B.); federica.brioschi@unimi.it (F.A.B.); 2 Ospedale Veterinario San Francesco, Via Newton 2, 20148 Milano, Italy; andrea.jacchetti@vetsanfrancesco.it

**Keywords:** alpha-2 agonists, anaesthetic drug combination, canine, cardiac function, cardiorespiratory effects, co-induction anaesthesia, intravenous anaesthesia, opioids, propodex, rapid anaesthesia induction

## Abstract

High induction doses of propofol (PPF) may cause adverse effects. Co-induction protocols can reduce doses and enhance the beneficial profile of each drug. This study compared the induction quality, clinical, and echocardiographic effects of two rapid co-inductions in healthy, unpremedicated dogs. Baseline cardiorespiratory and echocardiographic variables were recorded. Dogs randomly received rapid intravenous PPF (2.2 mg/kg) with either dexmedetomidine (3 µg/kg) (PROPODEX; *n* = 12) or butorphanol (0.4 mg/kg) (PROPOBUT; *n* = 12). Induction quality, additional PPF dose, intubation time, cardiorespiratory parameters (2, 5, 10, 15, 20 min), and recovery quality were recorded. A second echocardiography was performed 5 min after induction. Induction quality was significantly higher in PROPODEX, with significantly lower additional PPF requirements and shorter intubation time. In PROPODEX, heart rate significantly decreased from baseline and was significantly lower than PROPOBUT, while arterial blood pressures significantly increased in PROPODEX and decreased in PROPOBUT from baseline. PROPOBUT significantly reduced left ventricular (LV) diastolic volumes and increased E/A ratio, while PROPODEX significantly increased LV systolic diameter and volumes, and significantly decreased fractional shortening. Recovery was significantly faster and better in PROPODEX. PROPOBUT preserved cardiac function but caused hypotension. PROPODEX provided superior induction and recovery quality, and cardiorespiratory stability, with only mild systolic function depression.

## Simple Summary

Propofol is widely used for the induction of anaesthesia in dogs; however, high doses may cause adverse effects such as hypotension and respiratory depression. Co-administration with other agents can reduce the propofol requirement and improve safety. This study compared two induction protocols, propofol combined with dexmedetomidine and propofol combined with butorphanol, in healthy, unsedated dogs. The objectives were to assess how these combinations affected the quality and timing of induction and intubation, as well as the effects on cardiorespiratory variables and echocardiographic measurements. The propofol-dexmedetomidine combination resulted in faster and smoother inductions, required lower propofol doses, and enabled shorter intubation and recovery times compared with propofol-butorphanol. Moreover, dogs receiving propofol-dexmedetomidine maintained higher arterial blood pressures, although they experienced lower heart rates. Dogs given propofol-butorphanol showed lower blood pressures, but heart rates remained within normal sinus rhythm and close to baseline values. Echocardiographic measurements were only mildly affected in both groups and remained within clinically safe limits. Considering the smooth induction, the clinical stability, and the minimal depression of cardiac function, the use of propofol-dexmedetomidine may be a safe and effective option for quickly anaesthetizing healthy dogs without prior sedation.

## 1. Introduction

Balanced anaesthesia, widely adopted in clinical practice, involves the combined use of multiple pharmacologic agents to achieve and maintain an adequate anaesthetic plane. This multimodal approach was first proposed by Lundy in 1926 as an alternative to ether monotherapy [1]. Administering lower doses of several agents, rather than a higher dose of a single drug, allows anaesthetists to enhance therapeutic effects while minimizing drug-specific adverse effects [2,3].

Propofol (PPF) is a hypnotic agent widely used for the induction of general anaesthesia in dogs, valued for its rapid onset, quick and predictable recovery, and antiemetic properties [4]. In healthy, unpremedicated dogs, an intravenous bolus of 6–9 mg/kg typically enables tracheal intubation and transition to inhalation anaesthesia [4,5]. However, PPF is associated with dose-dependent respiratory depression, decreased systemic vascular resistance, and hypotension [6,7]. Consequently, it is often included in balanced protocols following premedication, which can reduce the induction dose by 20% to 80% in dogs [4,8]. When sedatives or analgesics are given together with induction agents, the approach is referred to as co-induction [9]. Several co-induction strategies have been explored in small animals [3,10]. Butorphanol (BUT) is a synthetic opioid with mixed agonist–antagonist activity, acting primarily as a κ-opioid receptor agonist while antagonizing μ-opioid receptors [11]. At clinical doses in dogs, it produces mild-to-moderate sedation and short-lasting analgesia, with minimal respiratory and cardiovascular depression [12,13]. These properties make it suitable for minor procedures or as part of multimodal protocols. When administered before PPF, BUT can reduce the induction dose [14,15] while maintaining heart rate and blood pressure and without exacerbating respiratory depression in dogs [14]. In humans, PPF-BUT co-induction has been associated with a lower incidence of respiratory side effects and apnoea, and with better hemodynamic stability compared with PPF-fentanyl [16] or PPF-ketamine [17]. Despite these promising findings, this co-induction protocol has received limited investigation in veterinary medicine and requires further study.

Dexmedetomidine is a highly selective α_2_-adrenergic agonist, with potent sedative, analgesic, and cardiovascular effects in dogs [18]. In human anaesthesia, only a few studies have evaluated DEX administered immediately prior to induction with PPF. Compared with PPF-fentanyl, this combination required a significantly lower PPF induction dose and provided superior attenuation of PPF-induced hypotension [19]. In addition, fewer apnoea episodes and improved postoperative pain scores have been reported [20]. In veterinary medicine, Groppetti et al. (2019) demonstrated that PPF-DEX co-induction in bitches undergoing elective caesarean section was safe for the dam and yielded excellent Apgar scores in the puppies [21]. However, this strategy has not been thoroughly characterised in unpremedicated, low-anaesthetic-risk dogs. Propofol has been shown to stimulate appetite and exert a direct antiemetic effect in dogs [22], whereas high doses of α_2_-adrenergic agonists have been associated with vomiting [23]. When administered without premedication, PPF at induction doses can cause prolonged apnoea and severe hypotension [24,25]. Conversely, DEX generally preserves respiratory drive [26], but induces an initial vasoconstriction-mediated hypertension followed by reflex bradycardia, in conjunction with the vagomimetic effect [26,27]. Furthermore, PPF lacks analgesic properties and may cause pain on injection [28], whereas DEX and BUT provide analgesia [13,27]. Based on these considerations, it was hypothesized that a co-induction protocol using reduced doses of both PPF and DEX would provide smooth induction, a stable anaesthetic plane, and good recovery, while maintaining favourable cardio-respiratory function, compared to PPF-BUT co-induction. PPF-DEX combination was also expected to limit adverse effects reported for each individual drug, such as hypotension, apnoea, excitement or hypertension, bradycardia, and vomiting. Concurrently, it was hypothesized that the PPF–BUT protocol would be associated with fewer echocardiographic alterations.

The aim of the present study was to investigate and compare the effects of rapid co-administration of PPF-DEX and PPF-BUT on induction time, induction quality, clinical variables, and recovery time and quality in healthy, unpremedicated dogs. Echocardiographic and electrocardiographic measurements obtained after both protocol administrations were also evaluated and compared.

## 2. Materials and Methods

The present study complies with ethical standards and was approved by the Institutional Ethical Committee for Animal Care at the University of Milan (OPBA_47_18/09/2020). Written informed consent was obtained from the owner of each dog prior to inclusion. Dogs underwent a pre-anaesthetic examination and were classified according to the American Society of Anesthesiologists (ASA) physical status system. Only ASA class I (normal healthy dogs with no detectable disease) or class II (slight or moderate disease causing no obvious incapacity) patients admitted to the Veterinary Teaching Hospital of the University of Milan for diagnostic or minor procedures (dental treatment and examination, radiography, otitis treatment/ear examination, anal sac treatment, and ophthalmic examination) were included in this prospective, randomized, blinded study. All dogs underwent complete haematological and biochemical analysis to confirm eligibility. Furthermore, eligible dogs were either healthy or affected by mild myxomatous mitral valve disease (MMVD) classified as stage B1 according to American College of Veterinary Internal Medicine (ACVIM) guidelines [29]. Exclusion criteria included a body condition score (BCS) > 6 (Nestle Purina Body Condition Scoring System, 1 [emaciated] to 9 [obese]) [30], abnormal chest conformation likely to interfere with echocardiographic measurements, and a nervous or aggressive temperament (Maddern temperament score > 2) [31] that prevented baseline echocardiography, arterial blood pressure measurement, or venous catheter placement. Moreover, dogs with ACVIM stages higher than B1 or with other cardiac diseases diagnosed during the baseline echocardiography were excluded from the study.

Food and water were withheld for eight and two hours, respectively, before the beginning of the study. Baseline parameters included heart rate (HR), respiratory rate (RR), non-invasive systolic (SAP), mean (MAP), and diastolic (DAP) arterial blood pressures, and rectal temperature. Heart rate was determined by auscultation with concurrent palpation of peripheral pulses, and RR by direct observation of thoracic excursions. Arterial blood pressures were measured using a high-definition oscillometric monitor (SunTech Vet30; SunTech Medical; Morrisville, NC, USA) with cuff selection according to the manufacturer’s instructions; the mean of three consecutive measurements was recorded. Rectal temperature was measured with a digital thermometer (Pic VedoFamily; Pikdare S.p.A., Bernate, Italy).

A baseline echocardiographic examination (bECHO) was performed by a single experienced echocardiographer using an ultrasonographic unit (Esaote MyLabFox Cardiovascular ultrasound scan; Esaote SPA, Genova, Italy) equipped with two multifrequency phased array probes and simultaneous ECG recording. All echocardiographic scans were performed with the dogs positioned in right and left lateral recumbency, following published guidelines [32]. Echocardiographic data were digitally stored and later analysed offline by the same cardiologist, blinded to treatment allocation and time point. Each echocardiographic measurement represented the mean of three consecutive cardiac cycles. Two-dimensional (2D)-guided M-mode measurements from the right parasternal short-axis view were used to obtain the left ventricular (LV) internal diameter in diastole (LVIDd) and systole (LVIDs) using the leading edge-to-leading edge method. Then, the left ventricular fractional shortening (FS%) was calculated as [33]:

FS% = (LVIDd − LVIDs/LVIDd) × 100(1)

The left atrial-to-aortic ratio (LA/Ao) was measured from the right parasternal short-axis view in B-mode, at the aortic valve level, in early diastole immediately after the aortic valve closure [34]. The LV volume in end-diastole (LVEDV) and end-systole (LVESV), as well as ejection fraction (EF; stroke volume/end-diastolic volume × 100), were obtained from the right parasternal four-chamber view in B-mode using Simpson’s method of discs (SMOD) [35]. Volumes were indexed to body surface area (EDVI: end-diastolic volume index; ESVI: end-systolic volume index), calculated as:

0.101 × body weight (kg)^2/3^. (2)

Stroke volume (SV) was calculated as the difference between LVEDV and LVESV, and cardiac output (CO) as SV multiplied by HR. Trans-mitral flow [E peak velocity (E-Vmax), A peak velocity (A-Vmax), E-Vmax to A-Vmax ratio (E/A)] was measured using pulsed-wave Doppler from the left apical four-chamber view. The aortic and pulmonary velocity time integrals (VTIAo and VTIPv, respectively) were determined from the subcostal view and right parasternal short-axis view, respectively. Lastly, mitral valve regurgitation was assessed by colour Doppler and graded as mild if the regurgitant jet area (ARJ) occupied less than 20–30% of the left atrium area (LAA; ARJ/LAA ratio), moderate if ARJ/LAA ratio was between 20–30% and 70%, severe if ARJ/LAA ratio was more than 70% [36].

A 22- or 20-gauge intravenous (IV) catheter (Jelco IV Catheter; ICU Medical; Roma, Italy) was aseptically placed and secured in the cephalic vein. Dogs were randomly assigned (www.randomizer.org) to either the PPF-DEX (PROPODEX) group or the PPF-BUT (PROPOBUT) group. All drugs were prepared into separate syringes by an experienced anaesthetist and labelled in a manner that did not reveal their content. Anaesthetic procedures were carried out by a different experienced anaesthetist, who was blinded to the treatment allocation. Dogs in the PROPODEX group received co-induction with PPF at 2.2 mg/kg (Proposure, 10 mg/mL, Merial Italia S.p.A., Milano, Italy) and DEX at 3 µg/kg (Dexdomitor 0.5 mg/mL; Vetoquinol Italia S.r.l., Bertinoro, Italy), whereas those in the PROPOBUT group were induced with PPF at 2.2 mg/kg and BUT at 0.4 mg/kg (Nargesic 10 mg/mL; ACME S.r.l., Cavriago, Italy). The time of induction was recorded as T0. In both groups, co-induction was administered manually via IV injection within 5 s, with DEX or BUT always given after PPF. The quality of anaesthetic induction was scored using a dedicated descriptive scale (Table 1).

Orotracheal intubation was performed once an adequate degree of unconsciousness was achieved, defined as the absence of voluntary movement, decreased palpebral reflex, adequate jaw relaxation, and no coughing attempts [37]. If required, additional IV boluses of PPF (0.5 mg/kg) were administered to achieve an adequate level of unconsciousness to obtain orotracheal intubation and to maintain general anaesthesia for 20 min following induction. The additional doses of PPF required for intubation (PPF-I) and for anaesthesia maintenance (PPF-M), as well as the time to intubation, were recorded.

Respiratory rate, peripheral oxygen saturation (SpO_2_), end-tidal carbon dioxide (ETCO_2_), HR, SAP, MAP, and DAP were continuously monitored and recorded at 2, 5, 10, 15, and 20 min from induction (T2, T5, T10, T15, T20, respectively). Peripheral oxygen saturation was measured using a pulse oximeter positioned on the tongue, which was connected to a multiparameter monitor (S5 Compact Anesthesia Monitor; Datex-Ohmeda, Madison, WI, USA); a mainstream capnometer (EMMA Capnograph; Masimo; Milano, Italy) was used for ETCO_2_ measurements from the time of intubation. At T5, post-treatment echocardiographic and electrocardiographic measurements (pECHO) were performed following the same methodology previously described.

Adverse effects observed during the anaesthetic period were recorded, including retching or vomiting, panting (>100 breaths/minute) [38], excitement, twitching or tremors, and cardiac arrhythmias. In accordance with current human guidelines [39], normocapnic dogs continuously monitored with capnography did not routinely receive supplemental oxygen; 100% oxygen administration was reserved for cases in which SpO_2_ decreased to ≤90%, or in the event of apnoea, defined as the absence of spontaneous respiratory effort for 30 s [40], in which case assisted ventilation was started. Additionally, SpO_2_ values < 95% prompted immediate verification of pulse oximeter probe placement, airway patency (e.g., partial obstruction of the tracheal tube, tracheal tube displacement), and spontaneous ventilation, according to AAHA anaesthesia and monitoring recommendations for dogs and cats [41].

The total anaesthesia time, defined as the interval from induction of general anaesthesia to the end of the procedure, was recorded. At the end of the procedures, the time from the end of the procedure to extubation, the time from extubation to sternal recumbency, and the time from sternal recumbency to standing were recorded for all dogs. Extubation criteria included the presence of strong palpebral reflexes and observation of two consecutive swallowing movements within a 10 s period [42]. Following extubation, dogs in the PROPODEX group received atipamezole (Antisedan 5 mg/mL; Vetoquinol Italia S.r.l., Bertinoro, Italy) at a dose of 30 µg/kg intramuscularly. Recovery quality and degree of ataxia were assessed using a standardized scoring system based on behavioral parameters; for recovery quality, scores were assigned as follows: 0 = excellent (smooth, calm, and uncomplicated), 1 = minimal vocalization and/or struggling, 2 = moderate vocalization and/or struggling, and 3 = poor (marked vocalization and struggling). For ataxia, scores were: 0 = none, 1 = minimal, 2 = moderate, and 3 = marked [43].

### Statistical Analysis

An a priori sample size calculation was performed considering a clinically relevant difference of 1 mg/kg in PPF dose required for orotracheal intubation, with an estimated standard deviation of 0.75 mg/kg [44]. This corresponded to an effect size of *d* = 1.333. Considering a study power of 80% and an α = 0.05, the sample size was estimated to be ten per group (G*Power©, v. 3.1, Franz Faul, Edgar Erdfelder, Albert-Georg Lang, and Axel Buchner, 2006, 2009, Germany). Statistical analysis was performed using IBM SPSS Statistics 26.0 (SPSS Inc., Chicago, IL, USA). The normality of data distribution was assessed by a Shapiro–Wilk test at the α = 0.05 level. Descriptive statistics were reported as mean ± standard deviation (SD) or median (range) for continuous and ordinal variables, respectively. Depending on normality, the *t*-test or the Mann–Whitney test was used to assess significant differences between groups in terms of body weight and age. Categorical variables (gender, ASA status, and number of dogs with valve regurgitation) were compared between groups using the Chi-square test. The total dose of PPF-I and PPF-M, intubation and recovery times, and quality of anaesthetic induction and recovery were compared between groups with the two-sided Student *t* test. Repeated-measures analysis of variance (ANOVA) and Friedman’s test were applied to cardiorespiratory variables to evaluate within-group differences over time. For comparisons between independent groups, one-way ANOVA was used. Echocardiographic measurements were compared using the Wilcoxon matched-pairs test for paired longitudinal comparisons within each group, and between-group comparisons were assessed with a *t* test for independent samples. A *p*-value < 0.05 was the limit of statistical significance.

## 3. Results

Twenty-four client-owned dogs were enrolled in the study. No statistically significant differences were detected between groups regarding age (PROPODEX 6.5 ± 1.8 years; PROPOBUT 5.1 ± 2.4 years), gender (PROPODEX 8 females, 4 males; PROPOBUT 5 females, 7 males), body weight (PROPODEX 25.5 ± 14.2 kg; PROPOBUT 23.1 ± 6.9 kg), and ASA status (PROPODEX ASA I: N = 5, ASA II: N = 7; PROPOBUT ASA I: N = 6, ASA II: N = 6). The quality of anaesthetic induction was significantly higher in the PROPODEX group (0; 0–1), in which eleven dogs received a score of 0 (smooth) and one dogs a score of 1 (fair), whereas in the PROPOBUT group, two dogs were scored as smooth (0) and ten dogs as fair (1) (1; 0–1; *p* = 0.0000). Furthermore, no dogs in the PROPODEX group required additional boluses of PPF-I and PPF-M, while those in the PROPOBUT group required 1.2 ± 0.8 mg/kg of PPF-I (*p* = 0.003) and 1.4 ± 1.4 mg/kg of PPF-M (*p* = 0.02). Time to intubation differed significantly between groups (*p* = 0.04), being shorter in the PROPODEX group (PROPODEX 75 ± 23 s; PROPOBUT 145 ± 67 s).

**Figure 1:**
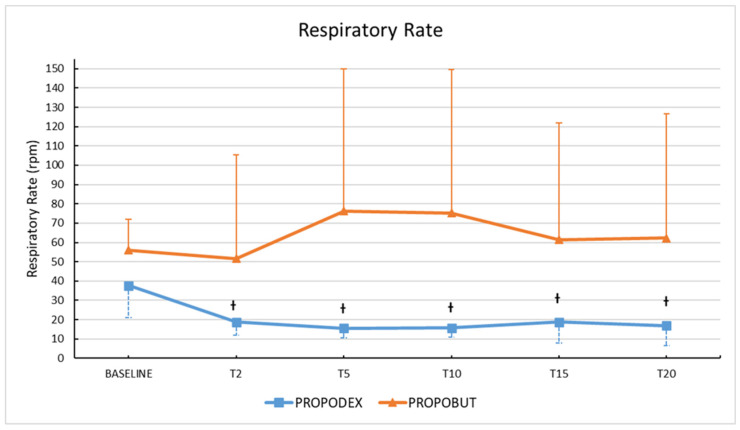
Mean (± standard deviation) respiratory rate in healthy unpremedicated dogs before and after the co-induction with propofol (2.2 mg/kg) and dexmedetomidine (3 µg/kg) (PROPODEX group; *n* = 12) or butorphanol (0.4 mg/kg) (PROPOBUT group; *n* = 12). Dogs were evaluated immediately before induction of general anesthesia (baseline) and at two minutes (T2), five minutes (T5), 10 min (T10), 15 min (T15), and at 20 min (T20) after co-induction administration. Significant differences in respiratory rate from baseline (represented with the symbol †, *p* ˂ 0.05) within the PROPODEX group were found.

No significant differences in RR were observed between groups. A significant reduction in RR from baseline value was observed within the PROPODEX group over time (*p* = 0.0001), as shown in Figure 1.

**Figure 2:**
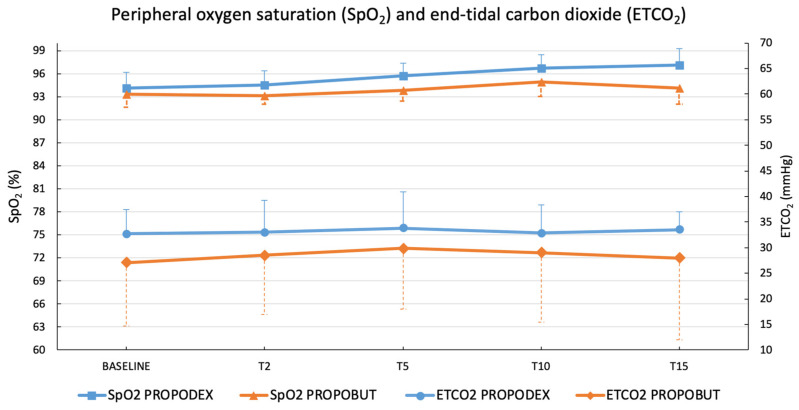
Mean (±standard deviation) peripheral oxygen saturation and end-tidal carbon dioxide in healthy unpremedicated dogs after the co-induction with propofol (2.2 mg/kg) and dexmedetomidine (3 µg/kg) (PROPODEX group; *n* = 12) or butorphanol (0.4 mg/kg) (PROPOBUT group; *n* = 12). Dogs were evaluated immediately before induction of general anesthesia (baseline) and at two minutes (T2), five minutes (T5), 10 min (T10), 15 min (T15), and at 20 min (T20) after co-induction administration. No significant differences between and within groups were found.

Peripheral oxygen saturation and ETCO_2_ were higher in the PROPODEX group than in the PROPOBUT group, but no significant differences were observed between groups and within groups over time. Results are shown in Figure 2. No patient in either group required supplemental oxygen or assisted ventilation, and no episodes of apnoea occurred during the procedures.

**Figure 3:**
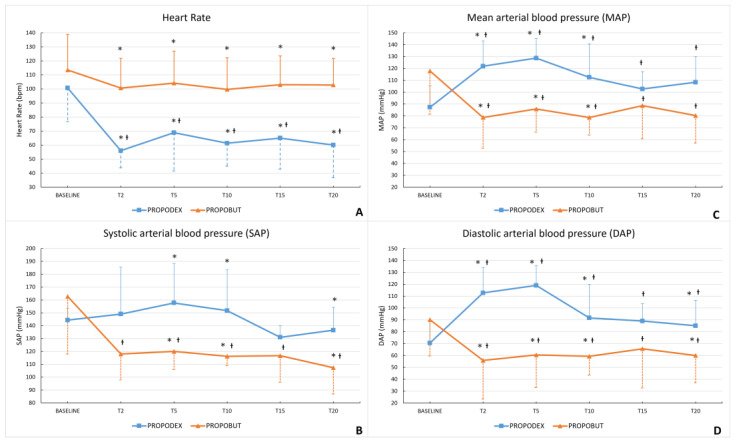
Mean (±standard deviation) heart rate (**A**), systolic (**B**), mean (**C**) and diastolic (**D**) arterial blood pressures in healthy unpremedicated dogs before and after the co-induction with propofol (2.2 mg/kg) and dexmedetomidine (3 µg/kg) (PROPODEX group; *n* = 12) or butorphanol (0.4 mg/kg) (PROPOBUT group; *n* = 12). Dogs were evaluated immediately before induction of general anesthesia (baseline) and at two minutes (T2), five minutes (T5), 10 min (T10), 15 min (T15), and at 20 min (T20) after co-induction administration. Significant differences (*p* < 0.05) between groups (represented as the symbol *) and within groups from baseline (represented as the symbol †) were found.

A reduction in HR from baseline was observed in all dogs included in this study, but this decrease reached statistical significance only in the PROPODEX group (*p* = 0.04). Furthermore, HR was significantly lower in the PROPODEX group than in the PROPOBUT group at all time points except the baseline (T2 *p* = 0.01; T5 *p* = 0.035; T10 *p* = 0.007; T15 *p* = 0.01; T20 *p* = 0.006). Results are summarized in Figure 3A. In the PROPOBUT group, SAP, MAP, and DAP showed a significant decrease from baseline over time (SAP *p* = 0.006; MAP *p* = 0.004; DAP *p* = 0.003), whereas in the PROPODEX group, MAP and DAP significantly increased from baseline (MAP *p* = 0.007; DAP *p* = 0.013); a non-significant increasing trend was also observed for SAP. Arterial blood pressures were significantly higher in the PROPODEX group compared with the PROPOBUT group at T2 (MAP *p* = 0.01; DAP *p* = 0.005), at T5 (SAP *p* = 0.02; MAP *p* = 0.002; DAP *p* = 0.001), at T10 (SAP *p* = 0.02; MAP *p* = 0.005; DAP *p* = 0.03), and at T20 (SAP *p* = 0.02; DAP *p* = 0.04) (Figure 3B–D), whereas no significant differences were observed at T15 between groups.

All echocardiographic results are displayed in Table 2.

In the PROPOBUT group, LVEDV and EDVI decreased significantly after drug administration (LVEDV *p* = 0.03; EDVI *p* = 0.03). In the PROPODEX group, significant increases in LVIDs, LVESV, and ESVI, along with a significant decrease in FS%, were observed (LVIDs *p* = 0.03; LVESV *p* = 0.03; ESVI *p* = 0.03; FS% *p* = 0.03). The E/A ratio was not influenced by PROPODEX administration, whereas in the PROPOBUT group it increased significantly at pECHO (*p* = 0.03). No statistically significant differences were detected between groups for any echocardiographic variable before and after drug administration. During bECHO, mild to moderate mitral valve regurgitation [36] was diagnosed in nine out of 12 dogs in the PROPODEX group. Specifically, six dogs presented mild mitral valve regurgitation (ARJ/LAA: 16.5%, 11–19%), while three dogs had moderate regurgitation (ARJ/LAA: 24%, 23–25%). At pECHO, a significant increase in regurgitation was observed, with ARJ/LAA ratio remaining below 30% (ARJ/LAA mild cases: 21%, 17–24%, *p* = 0.012; ARJ/LAA moderate cases: 29%, 27.0–29.0%, *p* = 0.007). In the PROPOBUT group, mild to moderate mitral valve regurgitation was detected in seven out of 12 dogs during bECHO. Particularly, three dogs had mild regurgitation (ARJ/LAA: 17%, 16.0–18.0%), while four dogs had moderate regurgitation (ARJ/LAA: 23.5%, 22.0–24.0%). At pECHO, the ARJ/LAA ratio significantly decreased in all affected dogs, and in two mild cases, regurgitation completely resolved, with ARJ/LAA of 0% (ARJ/LAA mild cases: 0%, 0–7%, *p* = 0.002; ARJ/LAA moderate cases: 9%, 7–10%, *p* = 0.0005). In dogs without mitral valve regurgitation at bECHO (3/12 dogs in the PROPODEX group and 5/12 dogs in the PROPOBUT group), no regurgitant area was detected even after induction of general anaesthesia.

No episodes of vomiting, excitement, twitching, tremors, or apnoea occurred in either group during the intra-anaesthetic period. Panting was observed in four dogs in the PROPOBUT group. No cardiac arrhythmias were recorded in the PROPOBUT group, whereas all dogs in the PROPODEX group developed synusal bradycardia following drug administration. Additionally, second-degree sino-atrial block and second-degree atrioventricular block were recorded in two and three dogs, respectively. Both arrhythmias were self-limited and resolved spontaneously in all cases between T10 and T15.

No significant difference was detected in total anaesthesia time between groups (PROPODEX: 19 ± 1.1 min; PROPOBUT 18 ± 1.4 min). Time from the end of the procedure to extubation (PROPODEX 4.9 ± 1.9 min; PROPOBUT 8.4 ± 3.3 min; *p* = 0.004), time from extubation to sternal recumbency (PROPODEX 3.7 ± 1.1 min; PROPOBUT 7.8 ± 3.5 min; *p* = 0.003), and from sternal recumbency to standing (PROPODEX 2.8 ± 1 min; PROPOBUT 5.1 ± 2.4 min; *p* = 0.006) significantly differed between groups. Recovery quality was significantly higher in the PROPODEX group (0, 0–1), with 11/12 dogs scoring 0 (excellent recovery), compared with the PROPOBUT group (1, 0–2), in which 3/12 dogs scored 0 (*p* = 0.001). Similarly, the PROPODEX group showed significantly lower ataxia scores than the PROPOBUT group (PROPODEX: 0, 0–1; PROPOBUT: 1, 0–2; *p* = 0.001). During the recovery, tremor/rigidity (four dogs), vocalization (one dog), and paddling (two dogs) were observed in the PROPOBUT group; all these events were short-lived and self-limiting, lasting less than two minutes.

## 4. Discussion

The present study aimed to evaluate and compare the anaesthetic, clinical, and echocardiographic effects of DEX and BUT for rapid co-induction of general anaesthesia with PPF in unpremedicated dogs. In veterinary literature, few studies have investigated pharmacological combinations for the induction of general anaesthesia in unsedated small animals [10,21,45,46]. To the author’s knowledge, this is the first study assessing co-induction with PPF and BUT in dogs; co-induction with PPF and DEX has been previously described by Groppetti and colleagues (2019) [21] but its cardiorespiratory and echocardiographic effects have never been evaluated.

In the present study, PPF-DEX combination resulted in a safe, smooth induction and rapid endotracheal intubation, without vomiting, likely reflecting preservation of PPF’s antiemetic effect [22]. Furthermore, this combination showed a pronounced sparing effect on the PPF dose required both for orotracheal intubation and for maintaining general anaesthesia for 20 min, as no patient required an additional hypnotic. These results align with the literature, as a marked synergism between α_2_-agonists and PPF has been reported in dogs [47,48]. Administration of α_2_-agonists induces deep sedation, muscle relaxation, and “background analgesia” by activating pre-synaptic α_2_-receptors in the central nervous system (CNS), thereby reducing central noradrenergic neurotransmission and the total amount of anaesthetics needed [18]. In healthy dogs, a dose-dependent PPF sparing effect has been demonstrated, with the extent depending on the IV DEX dose administered 5–15 min before induction [5,48,49]. Particularly, Kuusela et al. (2001) reported that the administration of 2 µg/kg of DEX given 10 min before induction reduced the PPF requirement to 2.7 ± 0.5 mg/kg [49]. Similar findings were observed in other studies [48], showing a 10–15% reduction in PPF dose with 2 µg/kg of DEX compared to 1 µg/kg [5,48]. In the present study, a similar dose of DEX (3 µg/kg) was used. Assuming a comparable dose-dependent reduction in PPF requirement, a starting dose of 2.2 mg/kg of PPF was selected. Based on the authors’ clinical experience, high-quality inductions can also be achieved with 2 µg/kg of DEX combined with a slightly higher PPF dose of 2.5 mg/kg. This suggests that general anaesthesia can be induced using different dose combinations of the same drugs, tailored to the desired clinical outcome, while accounting for their pharmacokinetics and pharmacodynamic properties. From a balanced anesthesia perspective, this principle should be considered in future studies evaluating the clinical safety of various pharmacological combinations. In humans, the significant PPF-sparing effect of DEX may be partly explained by competitive inhibition of the enzyme cytochrome CYP450, which is involved in the hepatic biotransformation and elimination of both drugs [50]. Comparable effects on induction quality and drug sparing effect were observed in the present study, possibly because DEX and PPF possess similar rapid onset and peak plasma concentration times in dogs [6,7,23]. However, the pharmacokinetics of these two drugs when administered in rapid succession have not been studied in dogs, and further investigations are needed to support this hypothesis.

The PPF-BUT combination yielded an induction of fair quality, likely because BUT was less effective than DEX in reducing the PPF dose needed. Dogs in the PROPOBUT group required an average of 2 or 3 additional PPF boluses to allow intubation, lengthening the overall intubation time, and to maintain general anaesthesia during the 20 min observation period. Previous studies reported that BUT has only mild-to-moderate effects on reducing the PPF induction dose [14] with a smaller impact than medetomidine in dogs [15]. This limited anaesthetic-sparing effect aligns with evidence that opioids lacking full μ-opioid receptor agonism generally provide a modest reduction in anesthetic requirements [51]. Furthermore, despite BUT may cause less dysphoria at clinical doses and is less likely to cause CNS excitement than full µ-opioid agonists, a rapid intravenous injection can cause a fast increase in plasma concentration, leading to transient excitement in several species [51]. Thus, despite its faster onset and greater sedative effect compared with other opioids [52], BUT may have contributed to the mild, transient excitement observed in most dogs in the PROPOBUT group.

Despite the rapid PPF administration in both groups, no patient developed apnoea or profound respiratory depression, which is typically reported at standard induction doses in unpremedicated dogs [24,25]. As previously described by Lerche and colleagues in 2004 [53], α_2_-agonists can cause a centrally mediated reduction in RR and minute ventilation, but preserving respiratory drive [26], leading to minimal or no increases in partial pressure of arterial carbon dioxide together with a mild reduction in partial pressure of arterial oxygen, changes not clinically relevant in healthy dogs [47,54]. Although arterial blood gas analysis was not assessed in the present study, in the PROPODEX group the significant RR reduction from baseline did not cause deviations in SpO_2_ and ETCO_2_ from physiological ranges, nor significant differences compared to the PROPOBUT group. Four dogs in the PROPOBUT group experienced panting following co-induction, probably due to the BUT, although this effect is uncommon compared to other opioids [52]. No dog in either group required supplemental oxygen, as SpO_2_ remained above 90% at all measurements, or assisted ventilation, because no episodes of apnoea occurred. While SpO_2_ values were slightly higher in the PROPODEX group, the difference was not statistically significant, indicating that both protocols could be considered acceptable options for procedural sedation (e.g., radiography, ultrasound examinations, auricular or nasal foreign body removal, and other minor interventions) in healthy dogs.

The cardiovascular effects observed in the present study matched the known effects of the anaesthetics used in each group and are consistent with previous studies carried out in dogs [48,49,55]. Indeed, PPF has a mild negative inotropic action, which may be mediated by inhibition of L-type Ca^++^ channels or modulation of Ca^++^ release from the sarcoplasmic reticulum, shifting the dose responsiveness to adrenergic stimulation and not altering the contractile reserve. However, the net effect of PPF on contractility is insignificant at clinical concentrations because of a simultaneous increase in the sensitivity of the myofilaments to activator Ca^++^ in humans [56]. Moreover, PPF blunts baroreflex responses and lowers arterial pressure via vasodilation [57]. It has been shown that DEX does not directly depress the canine myocardial function [58]; its bradycardia is initially attributable to an increase in peripheral vasoconstriction via α_2_B-receptor activation in peripheral vascular smooth muscles, in combination with an increase in vagal tone, and secondarily to central sympatholytic action [18]. The associated CO reduction is not mediated by decreased contractility but rather by the effects of an increased vascular resistance and decreased HR [57]. Dexmedetomidine can also induce first and second-degree atrioventricular blocks in dogs [59]. In contrast, BUT causes mild, dose-related decreases in blood pressure, HR, peak systolic pressure, and cardiac contractility when given intravenously [57]. Bradycardia is due to central vagal stimulation [57], while ventricular contractility but not atrial function [60] is directly depressed by k-opioid receptors acting on Ca^++^ channels in the right atrium and left ventricle [61]. Accordingly, in the PROPOBUT group, bradycardia was mild, and hypotension resulted from the combined effects of PPF and BUT, whereas, in the PROPODEX group, DEX counteracted PPF-induced hypotension, with bradycardia accompanied by a modest increase in arterial blood pressures and only self-limiting, vagally mediated arrhythmias.

Regarding the echocardiographic measurements, all values remained within the reference ranges [33,35,62], indicating that both protocols were safe in healthy and ACVIM B1 dogs. Both anaesthetic combinations produced minimal depression of cardiac function, only slightly reducing SV and CO. In both groups, left ventricular diameters decreased after co-induction except for LVIDs in the PROPODEX group, which increased significantly, likely due to increased vascular resistance [63]. A reduction in FS% was observed in both groups, reflecting decreased myocardial contractility, but it was more pronounced and statistically significant after DEX administration, as previously reported [63,64]. In the PROPOBUT group, hypotension probably reduced preload [65], leading to significant decreases in LVEDV and EDVI and a slight reduction in EF%. Although the preload reduction could have contributed to the observed increase in the E/A ratio [65], it can be speculated that neither protocol impaired left ventricular diastolic function. In contrast, in the PROPODEX group, increased afterload and decreased HR probably caused a mild reduction in systolic function, as indicated by increases in LVESV and ESVI, decreases in FS%, and a slight reduction in EF% [64]. Given these differing cardiovascular effects, PROPODEX may, albeit modestly, exacerbate pre-existing valvular regurgitation, whereas PROPOBUT may reduce or mask regurgitant jets, as observed in the present study. Therefore, when sedation is planned for diagnostic cardiac imaging, the choice of protocol could lead to overestimation (with PROPODEX) or underestimation (with PROPOBUT) of the severity of valvular disease. Such misinterpretation could directly influence diagnostic accuracy, treatment decisions, and prognosis. For these reasons, close collaboration between the cardiologist and the anesthetist is essential to select the most appropriate sedation plan, balancing hemodynamic stability with diagnostic reliability. Veterinary research on the relationship between anesthetic protocols and echocardiographic assessment is expanding [66], and the present findings highlight the need to integrate these considerations into both routine clinical practice and the design of future studies. The present study included only healthy dogs classified as ASA I-II (including ACVIM stage B1). However, in clinical practice, anaesthesia may also be required in patients with significant comorbidities (ASA ≥ III), including those with MMVD at ACVIM stage ≥ B2. In such cases, PROPODEX could increase the regurgitant area, potentially leading to severe adverse effects (e.g., pulmonary oedema). Conversely, PROPOBUT may limit the regurgitant area and thereby improve the hemodynamic outcome of these patients. However, additional studies are warranted to determine the extent to which these two protocols influence the hemodynamic status of patients classified as ASA ≥ III.

Recovery quality and degree of ataxia were assessed using a standardized behavioral scoring system as previously described by Hampton et al. (2019) [42]. The PROPODEX group exhibited significantly shorter recovery times than the PROPOBUT group, accompanied by superior recovery quality and a significantly lower degree of ataxia. In the PROPODEX group, no additional PPF-I or PPF-M was required; thus, the co-induction PPF dose may have been fully metabolized by the time of recovery [4], minimizing residual drug effects. In contrast, several dogs in the PROPOBUT group required additional PPF-I and PPF-M, which may have led to residual drug plasma concentrations at the time of recovery, potentially contributing to poorer recovery quality and causing paddling and tremors. However, further pharmacokinetic investigations are warranted to confirm this hypothesis. Moreover, in the PROPODEX group, intramuscular administration of atipamezole immediately after extubation antagonized the effects of DEX, further accelerating recovery time and significantly reducing ataxia by limiting residual sedation and motor impairment [54]. Therefore, these two factors likely acted synergistically to produce faster, higher-quality, and more coordinated recoveries in the PROPODEX group.

The main limitations of this study include the small sample size, which may have limited the detection of subtle differences between groups, and the absence of arterial blood gas analysis to confirm the lack of respiratory depression. Further studies with larger populations and comprehensive respiratory monitoring are needed to better evaluate the safety, efficacy, and analgesic effects of DEX and BUT in combination with PPF for rapid co-induction in unpremedicated veterinary patients.

## 5. Conclusions

In conclusion, both PROPODEX and PROPOBUT were safe and effective for rapid co-induction of general anaesthesia in healthy dogs, without clinically relevant cardiorespiratory or echocardiographic changes. However, clear clinical differences emerged between the groups. PROPODEX provided higher-quality induction, rapid orotracheal intubation, avoided supplemental PPF, and, with atipamezole reversal, resulted in faster, higher-quality recovery and lower ataxia scores compared with PROPOBUT. PROPOBUT often required additional PPF, leading to longer recovery times and transient adverse events. Cardiovascular effects reflected the known agents’ pharmacodynamic profiles: DEX countered PPF-induced hypotension by a modest increase in systemic vascular resistance but caused bradycardia, while PROPOBUT was associated with mild hypotension and preserved sinus rhythm. Both minimally affected cardiac function, but their hemodynamic differences may influence valvular regurgitation assessment, highlighting the need for collaboration between anaesthetists and cardiologists when designing sedation protocols for echocardiographic evaluations. Overall, both co-induction protocols are particularly suitable for minor procedures and/or diagnostic investigations in unpremedicated healthy dogs, with PROPODEX with reversal offering a more clinically advantageous option for ultra-short anaesthesia.

## Figures and Tables

**Table 1:** Scoring system used to evaluate the quality of anaesthesia induction after the rapid intravenous administration of propofol (2.2 mg/kg) and dexmedetomidine (3 µg/kg) or butorphanol (0.4 mg/kg) in unpremedicated dogs.

Scores	Quality of Anaesthesia Induction
0 (smooth)	Patient safely assumed sternal decubitus Induction occurred gradually and quietly without excitement Presence of ventro-rotation of the eyeball No periods of apnoea were observed Orotracheal intubation was possible.
1 (fair)	Patient assumed sternal or lateral decubitus Slight excitement, muscle twitching, or mild and transient limb movement No ventro-rotation of the eyeball Less than 30 s periods of polypnoea or apnoea were observed Orotracheal intubation required additional propofol to be achieved.
2 (poor)	Patient was ataxic Marked excitement, myoclonus, or prolonged paddling, and possible vocalizations No ventro-rotation of the eyeball More than 30 s periods of polypnoea or apnoea were observed Orotracheal intubation required additional propofol to be achieved

**Table 2:** Mean (±standard deviation) echocardiographic variables in healthy unpremedicated dogs before (bECHO) and after (pECHO) the co-induction with propofol (2.2 mg/kg) and dexmedetomidine (3 µg/kg) (PROPODEX group; *n* = 12) or butorphanol (0.4 mg/kg) (PROPOBUT group; *n* = 12).

Variable	Time	PROPODEX	PROPOBUT
		Mean ± SD	Mean ± SD
**LVIDd (mm)**	bECHO	37.38 ± 11	39.7 ± 5.82
	pECHO	36.75 ± 12.17	37.18 ± 5.09
**LVIDs (mm)**	bECHO	22.22 ± 8.36 ^†^	26.2 ± 4.88
	pECHO	25.7 ± 9.03 ^†^	25.2 ± 4.77
**FS (%)**	bECHO	41 ± 8.83 ^†^	34 ± 6.7
	pECHO	29.83 ± 7.08 ^†^	32.5 ± 6.44
**LVEDV (mL)**	bECHO	50.88 ± 37.44	55.6 ± 27.9 ^†^
	pECHO	58.4 ± 42.1	45.43 ± 22.4 ^†^
**LVESV (mL)**	bECHO	19.65 ± 16.08 ^†^	21.32 ± 10.97
	pECHO	32.3 ± 25.73 ^†^	20.22 ± 12.71
**EDVI (mL/m^2^)**	bECHO	50.26 ± 24.23	63.98 ± 19.55 ^†^
	pECHO	59.47 ± 25.13	49.22 ± 15.47 ^†^
**ESVI (mL/m^2^)**	bECHO	19.13 ± 11.18 ^†^	22.82 ± 8.87
	pECHO	35.51 ± 16.9 ^†^	21.24 ± 9.43
**EF (%)**	bECHO	62.5 ± 11.47	64.5 ± 8.07
	pECHO	46 ± 14.91	58.5 ± 7.74
**LA/Ao**	bECHO	1.25 ± 0.18	1.20 ± 0.17
	pECHO	1.38 ± 0.35	1.15 ± 0.19
**SV (mL)**	bECHO	31.25 ± 22.41	34.33 ± 19.48
	pECHO	26.1 ± 19.8	25.23 ± 10.02
**CO (L/min)**	bECHO	2.34 ± 1.83	3.52 ± 2.32
	pECHO	1.91 ± 1.58	2.66 ± 1.19
**E-Vmax (m/second)**	bECHO	0.62 ± 0.11	0.75 ± 0.15
	pECHO	0.67 ± 0.22	0.71 ± 0.13
**A-Vmax (m/second)**	bECHO	0.62 ± 0.16	0.7 ± 0.24
	pECHO	0.54 ± 0.15	0.52 ± 0.11
**E/A**	bECHO	1.04 ± 0.22	1.19 ± 0.41 ^†^
	pECHO	1.25 ± 0.30	1.43 ± 0.44 ^†^
**VTIPv (cm)**	bECHO	0.14 ± 0.03	0.15 ± 0.02
	pECHO	0.12 ± 0.03	0.13 ± 0.03
**VTIAo (cm)**	bECHO	0.17 ± 0.03	0.16 ± 0.02
	pECHO	0.14 ± 0.03	0.13 ± 0.02

A-Vmax, A-wave peak velocity; CO, cardiac output; E/A, E-wave peak velocity to A-wave peak velocity ratio; EDVI, end-diastolic volume indexes; EF%, ejection fraction; ESVI, end-systolic volume indexes; E-Vmax, E-wave peak velocity; FS%, fractional shortening; LA/Ao, left atrial-to-aortic ratio; LVEDV, left ventricular end diastolic volumes; LVESV, left ventricular end systolic volumes; LVIDd, left ventricular internal diastolic diameters; LVIDs left ventricular internal systolic diameters; SV, stroke volume; VTIAo, aortic velocity time integral; VTIPv, pulmonary velocity time integral. Significant differences within groups (represented as the symbol ^†^, *p* ˂ 0.05) were found.

## Data Availability

The original contributions presented in this study are included in the article. Further inquiries can be directed to the corresponding author(s).

## Abbreviations

The following abbreviations are used in this manuscript:

2-D	Two-dimensional
ANOVA	Repeated-measures analysis of variance
AO	Aortic diameter
ARJ	Regurgitant Jet Area
ARJ/LAA ratio	Regurgitant Jet Area to Left Atrium Area Ratio
ASA	American Society of Anaesthesiologists
A-V max	A peak velocity
BCS	Body condition score
BPM	Beats per minute
BSA	Body surface area
bECHO	Baseline echocardiography
BUT	Butorphanol
CNS	Central nervous system
CO	Cardiac output
DAP	Diastolic arterial pressure
DEX	Dexmedetomidine
E/A	E peak velocity to A peak velocity ratio
EDVI	End diastolic volume index
EF	Ejection fraction
ESVI	End-systolic volume index
ETCO_2_	End-tidal carbon dioxide
E-V max	E peak velocity
FS	Fractional shortening
HR	Heart rate
IV	Intravenous
LA	Left atrial
LA/Ao	Left atrial-to-aortic ratio
LAA	Left atrium area
LV	Left ventricular
LVEDV	Left ventricular end diastolic volume
LVESV	Left ventricular end-systolic volume
LVIDd	Left ventricular internal diastolic diameter
LVIDs	Left ventricular internal systolic diameter
MAP	Mean arterial pressure
pECHO	Post-induction echocardiography
PPF	Propofol
PPF-I	Propofol required for intubation
PPF-M	Propofol required for maintenance
PROPOBUT	Propofol-butorphanol group
PROPODEX	Propofol-dexmedetomidine group
RPM	Rest per minute
RR	Respiratory rate
SAP	Systolic arterial pressure
SD	Standard deviation
SMOD	Simpson’s method of discs
SpO_2_	Peripheral oxygen saturation
SV	Stroke volume
T0	Baseline time (induction time)
T2	Two minutes from induction
T5	Five minutes from induction
T10	Ten minutes from induction
T15	Fifteen minutes from induction
T20	Twenty minutes from induction
VTD	Telediastolic volume
VTIAo	Aortic velocity time integral
VTIPv	Pulmonary velocity time integral
